# Supplementary material for: Addition of Probiotics to Anti-Obesity Therapy by Percutaneous Electrical Stimulation of Dermatome T6. A Pilot Study
Source: Int J Environ Res Public Health. 2020 Oct 3;17(19):7239. doi: 10.3390/ijerph17197239 (PMC7579484; doi:10.3390/ijerph17197239)
Supplement: Supplementary file 1 [file ijerph-17-07239-s001.pdf]

International Journal of
Environmental Research and Public Health

MDPI

Supplementary Materials: Addition of Probiotics to Anti-Obesity Therapy by Percutaneous Electrical Stimulation of Dermatome T6. A Pilot Study

BACTERIA	N	Media (NGC/g)	Median Percentile 50 (NGC/g)	Median Percentil 10 (NGC/g)	Median Percentil (NGC/g)	Median Percentile 75 (NGC/g)	Median Percentile 90 (NGC/g)	Reference Value(NGC/g)
Actinobacteria	1,533	7.54	8.05	4.49	6.56	8.97	9.66	**6.50-9.00**
Akkermansia muciniphila	1,546	5.48	6.30	1.80	3.20	7.60	8.20	**5.00-8.50**
Bacilus spp	487	2.59	2.70	1.20	2.20	3.30	3.70	**0.00-4.00**
Bacteroides spp	1,546	8.46	8.60	7.50	8.10	9.00	9.30	**7.00-8.50**
Bacteroidetes	1,546	8.76	8.83	7.74	8.29	9.32	9.65	**8.00-10.00**
Bifidobacterium spp	1,546	6.56	7.00	3.90	5.70	7.80	8.40	**5.00-7.00**
C. Ccoccoides	1,533	8.31	8.50	7.40	8.00	8.90	9.30	**7.00-9.00**
C. Perfringens	1,533	4.66	4.80	3.35	4.20	5.30	5.80	**0.00-5.50**
Campylobacter spp	1,533	0.79	0.00	0.00	0.00	0.00	4.30	**0.00-3.50**
E. Coli	1,546	4.48	4.60	2.00	3.40	5.70	6.70	**4.00-6.50**
Enterobact. (exc. E.coli)	1,546	2.07	1.60	0.60	1.00	2.60	4.10	**0.00-2.50**
Enterococos spp	1,546	6.54	6.50	5.30	5.90	7.20	8.00	**6.00/8.50**
F. Prausnitzii	1,546	7.52	7.60	6.30	7.00	8.10	8.60	**6.50/8.00**
Firmicutes	1,546	9.00	9.04	8.25	8.64	9.40	9.67	**8.50-10.00**
Helicobacter spp	1,533	1.32	0.00	0.00	0.00	2.70	3.40	**0.00-4.00**
Lactobacillus spp	1,541	5.02	4.90	3.75	4.30	5.70	6.40	**4.50-7.00**
Prevotella spp	1,546	6.55	6.20	4.60	5.20	8.20	9.00	**5.00-8.50**
Proteobacteria	1,533	5.6	5.68	3.71	4.72	6.63	7.42	**3.00-7.00**
Pseudomona spp	1,533	1.18	0.00	0.00	0.00	2.20	2.90	**0.00-4.00**
Saccaromyces cereviseae	1,533	3.22	3.30	2.10	2.70	3.80	2.40	**2.00-5.00**
Staphilococus spp	1,533	3.02	3.00	2.10	2.50	3.50	4.00	**2.50-5.00**
Verucomicrobia	1,533	5.98	6.80	1.94	3.46	8.21	8.86	**5.50-9.00**
Muconutritive microbiota	1,533	8.03	8.10	7.20	7.70	8.50	8.80	**7.00-9.00**
Immunomodulatory microbiota	1,533	7.53	7.60	6.20	6.90	8.20	8.70	**6.50-8.50**
Proteolitic microbiota	1,533	8.54	8.60	7.70	8.20	9.00	9.40	**6.50-9.00**
RATIOS								
Firmicutes/Bacteroidetes	1,546	0.24	0.23	`-0.38	`-0.07	0.53	0.84	**`-0.10/0.30**
Bacteroides/Prevotella	1,546	1.91	1.80	`0.20	0.40	3.30	4.00	**0.20/3.10**
C. Coccoides/C.perfringens	1,527	3.67	3.70	2.60	3.20	4.30	4.90	**1.50/5.50**
Enterobact./Enterococcus	1,546	0.01	`0.03	`-2.18	`-1.13	1.05	2.12	**`-1.00/2.00**
Reoseburia spp/Eubacterium	1,533	7.23	7.30	6.40	6.90	7.70	8.00	**6.50/8.50**

Figure S1. Gut bacteria in control subjects. Fecal samples from one hundred age- and sex-matched non-obese, normoglycemic and normolipidemic voluntaries without known cardiovascular, malignant and digestive diseases were analyzed to estimate the control ranges for each bacteria. NGC/g: number of gene copies per gram of feces.
